# Femoral Epiplocele in a Woman—Radical Operation

**Published:** 1894-11

**Authors:** William J. Taylor


Femoral Epiplocele in a Woman; Radical Operation. By William J. Taylor, M. D.—I wish to present a specimen of omental hernia, removed to-day with the assistance of Dr. T. S. K. Morton. The history is briefly as follows:

A woman, aged forty-six years, had first noticed a lump in her right groin twelve years ago, but it gave no trouble until about four or five months ago, when it enlarged and became very painful. She was in the country at the time, and went to bed and applied poultices to the swelling. In a few days she was much improved, the pain and swelling relieved, but the mass did not disappear entirely. She came to town, and I saw her first ten day ago. There was a good deal of swelling above Poupart's ligament and in the inguinal canal on the right side. I was in doubt whether it was a new growth or a hernia. With rest in bed and care the amount of inflammatory thickening was very much reduced. To-day I made an incision over the tumor, and came down upon a very hard tense sac, which easily dissected away, and I came upon this mass just above the internal inguinal ring. I then opened the sac, and drew out the omentum until I came to a healthy portion, and put a ligature around it and removed this mass. The sac was dissected free from the adhesion and ligated with silk. The whole mass showed evidence of long-standing inflammation. When I came close to the abdominal ring I discovered that it was a femoral hernia. I drew the edges of the canal together with two sutures, without injury to the artery; during the opertion only one small vessel required ligation. The specimen presented is the mass of omentum, which is glued together by inflammatory adhesion, and a portion of the sac.

